# Unusual complication of aortic dissections: intimo–intimal intussusception

**DOI:** 10.5830/CVJA-2015-029

**Published:** 2015

**Authors:** Unsal Vural, Ahmet Yavuz Balci, Ahmet Arif Aglar, Mehmet Kizilay, İbrahim Yekeler, Abdullah Kemal Tuygun

**Affiliations:** Dr Siyami Ersek Thoracic and Cardiovascular Surgery Center, Istanbul, Turkey; Dr Siyami Ersek Thoracic and Cardiovascular Surgery Center, Istanbul, Turkey; Dr Siyami Ersek Thoracic and Cardiovascular Surgery Center, Istanbul, Turkey; Dr Siyami Ersek Thoracic and Cardiovascular Surgery Center, Istanbul, Turkey; Dr Siyami Ersek Thoracic and Cardiovascular Surgery Center, Istanbul, Turkey; Dr Siyami Ersek Thoracic and Cardiovascular Surgery Center, Istanbul, Turkey

**Keywords:** intimo-intimal intussusception, complication of aortic dissections, DeBakey type I and type II, surgical treatment, urgent surgical intervention

## Abstract

Angiography with a pre-diagnosis of acute coronary syndrome was performed in a 76-year-old female patient presenting to another hospital with symptoms of chest pain and syncope. Upon determination of type III aortic dissection, the patient was referred to our clinic. On CT angiography, the ascending aortic diameter was 57 mm and no dissection flap was observed. There was a filling defect suggestive of intimo–intimal intussusception at the level of the aortic arch, occlusion of the left arteria carotid communis, and a double-channel aorta extending from the left subclavian artery to the iliac artery. On transoesophageal echocardiography, the ascending aorta was seen to be larger than normal and no dissection flap was observed. There were findings suggestive of haematoma and intimo–intimal intussusception at the proximal part of the aortic arch. The dissection flap causing occlusion in the vascular structures was resected. Supracoronary graft replacement of the ascending aorta was performed. Transoesophageal echocardiography is an invasive investigative method with high sensitivity and specificity for the diagnosis of intimo–intimal intussusception.

## Abstract

Intimo–intimal intussusception is a rare but fatal complication of aortic dissection. Emergency surgery is a life-saving procedure in aortic dissection. Intimo–intimal intussusception is an atypical manifestation of aortic dissection produced by circumferential dissection of the media and intimal layer of the aorta and invagination of the intimal flap in the aortic arch in DeBakey type I and type II aortic dissections.

Due to the absence of the intimal flap and crescent sign in the ascending aorta, it is difficult to diagnose this condition. A definitive diagnosis is made on transoesophageal echocardiography (TEE) with the observation of a prolapse of the dissection flap into the ascending aorta. Delaying the diagnosis may result in delayed treatment and increased risk of mortality. We present our case with a literature review of diagnosis and treatment of intimo-intimal intussusception.

## Case report

Angiography was performed for a pre-diagnosis of acute coronary syndrome in this case presenting to a cardiology centre with symptoms of syncope and chest pain lasting for three days. She was referred to our clinic with a pre-diagnosis of type III aortic dissection since a catheter had been placed into the false lumen and it could not be advanced into the aortic arch.

The 76-year-old female patient was admitted for the purpose of investigation and treatment. It was learned from her history that she had had hypertension for 10 years, and had been receiving treatment for 15 years for diabetes mellitus. On physical examination, she had poor consciousness with a poor general condition and a tendency to sleepiness. Her blood pressure and pulse rate were determined at 180/100 mmHg and 120 beats/min, respectively. Her left radial pulse was absent.

While the rhythm on a 12-lead ECG tracing taken three days earlier was normal sinus rhythm, the rhythm was ‘atrial fibrillation’ on the subsequent ECG. There was no finding suggestive of coronary ischaemia. Troponin T levels were within the normal ranges (troponin T = 0.05 ng/ml). Echocardiography was reported as ‘left ventricular functions were normal, the ascending aorta and the aortic root were larger than the normal diameter of the aorta, a mild aortic valve regurgitation was present, pericardial effusion was absent, no dissection flap was determined in the ascending aorta’.

**Figure 1.**
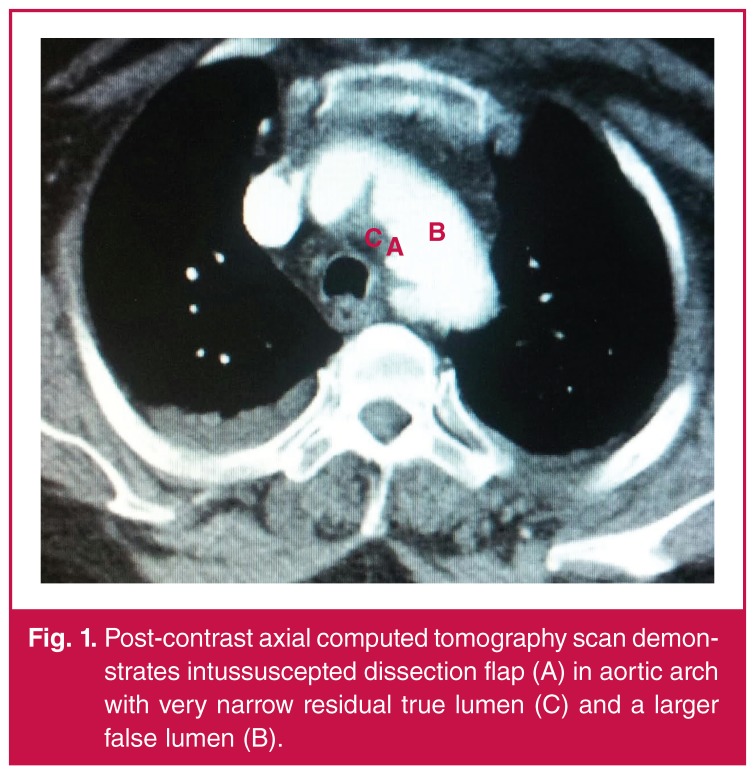
Post-contrast axial computed tomography scan demonstrates intussuscepted dissection flap (A) in aortic arch with very narrow residual true lumen (C) and a larger false lumen (B).

On CT angiography, the ascending aortic diameter was 57 mm and no dissection flap was present. A filling defect suggestive of intimo–intimal intussusception was observed at the level of the aortic arch. The left arteria carotid communis was occluded from the left subclavian artery. A double-channel aorta extending to the iliac artery was observed in the thoracic and abdominal aorta (Fig. 1).

On TEE, the ascending aorta was seen to be larger than normal and no dissection flap was observed. There was the appearance of a haematoma at the proximal part of the aortic arch and a flap entering into the aorta from the aortic arch during ventricular diastole. Mild aortic valve regurgitation due to dilation was confirmed.

A filling defect had been determined at the level of the aortic arch on the angiography performed in another centre. It was learned that the angiography could not be continued since the catheter had been placed into the false lumen. Considering the poor condition of the patient and the possibility of rupture of the dissection, she was operated on emergently.

## Surgical treatment

A median sternotomy was performed under general anaesthesia. Arterial cannulation was performed through a 10-mm graft anastomosed to the right subclavian artery in an end-toside fashion. Venous cannulation was performed through the right atrium and cardiopulmonary bypass was established. Left ventricular decompression was achieved through the right upper pulmonary vein.

Total circulatory arrest (TCA) was established by reducing the temperature to approximately 18°C. Iced saline was applied around the patient’s head for brain protection. Cerebrovascular circulation was established by administering one-fifth of the total cardiac output to the right carotid artery through the right subclavian artery during circulatory arrest. Coronary perfusion was established with antegrade blood cardioplegia.

**Figure 2.**
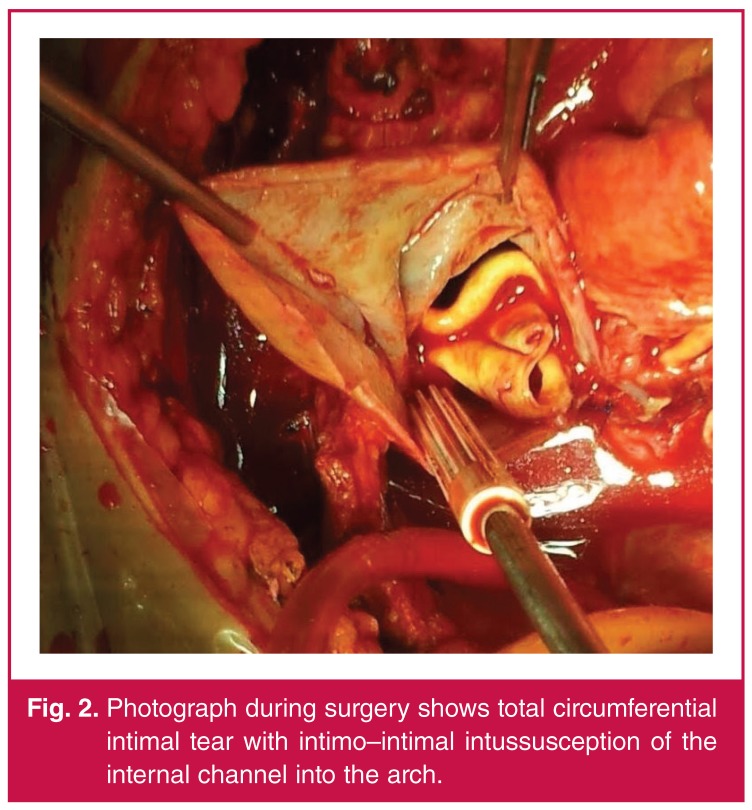
Photograph during surgery shows total circumferential intimal tear with intimo–intimal intussusception of the internal channel into the arch.

The ascending aorta was opened in an oblique fashion. It was observed that the intimal layer of the ascending aorta caused an obstruction by prolapsing into the aortic arch (Fig. 2). A definitive diagnosis was made by observation of the prolapse of the circumferential dissection flap into the aortic arch. The prolapsed intimal layer flap was resected through the aortic arch. The coronary ostia were seen to be open and retrograde flows were sufficient in the aortic arch.

The distal part of the ascending aorta was constructed using a 30-mm Dacron graft (Gorotex®) with a continuous suture technique. The posterior part of the anastomosis was supported with single-pledget mattress sutures. After draining the air bubbles in the artery, the ascending aorta was cross-clamped to the new vascular graft and cardiopulmonary bypass was established. Deep hypothermia was terminated. TCA lasted 13 minutes. The function and structure of the aortic valve and coronary ostia were normal.

Fibrin glue was used to adhere the dissection flap at the proximal anastomosis site of the graft in the supracoronary region. The flap was sutured over itself with a continuous suture technique. The Dacron graft (Gorotex®) was anastomosed to the supracoronary aorta with a continuous suture technique. The posterior part of the anastomosis was supported with pledget mattress sutures. The aortic cross-clamp was terminated after draining the air bubbles in the heart and the new aorta. Cardiopulmonary bypass was terminated after normothermia. After bleeding was controlled, the tissues were closed.

The patient was discharged after one day of postoperative intensive care and 12 days of follow up. During hospitalisation, antihypertensive and prophylactic antibiotic therapies were administered. Lifelong antihypertensive treatment was recommended. No problem was determined at the first month’s postoperative follow up. After six months, it was observed that the false lumen was closed on CT angiography

## Discussion

A dissection of the ascending aorta is only rarely circumferential. Complete circular dissection of the aorta was reported for the first time by Bostroem in 1887.^1^ Hufnagel named this complication intimo–intimal intussusception.^2^ Intussusception of the internal cylinder in the external aortic cylinder during circumferential dissection may induce obstruction of the aortic lumen or obstruction of the ostia of the supra-aortic vessels.^3^

Typical features of intimo–intimal intussusception are acute onset of chest or back pain. Goldberg *et al.* reported that when the cerebral vascular bed was affected, adverse neurological events were reported in 5–10% of cases.^4^ As in our case, when the subclavian artery was affected, asymmetric upper extremity blood pressures occurred.^5^ When the spinal canal and peripheral nerves are affected, symptoms such as paresis and plegia occur.^6^

One of the most important factors in making a diagnosis of aortic dissection is a high index of suspicion. Usually physical examination leads to a diagnosis of suspicion. Hypertension is either the main reason for dissection or it develops secondary to severe pain. Hypotension is an important finding of tamponade or coronary flow impairment.

Since chest pain was followed by unconsciousness in the history of our patient with hypertension, our pre-diagnosis was aortic dissection. Absence of a left radial pulse on physical examination strengthened the diagnosis of aortic dissection. Absence of flow in the left common carotid and subclavian artery was confirmed with CT angiography. TEE revealed that the absence of flow was due to intimo–intimal intussusception. During surgery, it was observed that the cause of occlusion was a dissection flap prolapsing into the aortic arch.

Suspicion of a diagnosis of intimo-intimal intussusception is life saving. On CT angiography, the intimal flap, false lumen and crescent sign in the ascending aorta observed in DeBakey type I and type II aortic dissections are absent in these patients. As in our case, a filling defect may be determined in the aortic arch by prolapsing of the intima (Fig. 1). ‘False occlusions’ may be observed in the vascular structures of the brain and extremities. Delay in treatment causes transformation of the occlusion to a ‘real occlusion’.^7^ There was no evidence of dissection of the ascending aorta in our case. TEE confirmed the haematoma in the distal part of the ascending aorta and motion of the intimal flap towards the ascending aorta during diastole.

Lajevardi *et al.* and Nohara *et al.* reported that the proximal part of the circumferential dissection in the ascending aorta occluded the coronary ostia and caused severe aortic valve insufficiency by entering the aortic valve.^3^,^8^ Aortic valve insufficiency due to a dissection flap is present in 60–70% of type I dissections.^9^ This condition occurs with three mechanisms of action: (1) central regurgitation due to annular dilation, (2) distortion of aortic root geometry due to prolapse of the dissection across a leaflet, (3) annulus rupture or tearing of one of the leaflets.^10^

Sometimes aortic valve insufficiency can be the only finding suggestive of dissection of the ascending aorta in patients without symptoms of dissection.^10^ In our case, aortic valve insufficiency was considered to be due to annular dilation. Cases resulting in a fatal outcome due to occlusion in the coronary ostia during diastole, caused by the proximal part of the dissection flap, have also been reported.^3^ Myocardial infarction is seen at a rate of 1–2% in aortic dissections.^11^

The dissection flap in the coronary ostia can be seen during angiography, with a diagnosis of coronary syndrome, as in our case.^11^ A diagnosis of aortic dissection must be excluded in acute coronary syndrome, otherwise antiplatelet and anticoagulant therapy may have fatal outcomes. Angiography was also performed in our case, with a diagnosis of acute coronary syndrome. Not being able to advance the angiography catheter into the aortic arch and determination of the false lumen caused a misdiagnosis of type III aortic dissection. Since angiography could not be completed, the coronary ostia could not be seen.

During surgery, we determined that the coronary ostia were occluded by the distal part of the dissection flap during diastole. In the literature, cases have frequently been reported of patients misdiagnosed with type III aortic dissection when the dissection flap was not observed in the ascending aorta.^12^

Where other diagnostic methods are insufficient in the diagnosis, TEE may be helpful, as the motion of the dissection flap invaginated in the aortic arch can be observed. Due to motion of the proximal part of the dissection flap toward the ventricle, it may be observed that it causes aortic valve insufficiency. TEE is considered to be the reference investigation with 98% sensitivity.^8^,^12^ TEE was also the most powerful diagnostic method in our case.

## Conclusion

Intimo–intimal intussusception is a rare complication of aortic dissection. However, it may be severe or fatal due to its effect on the cerebral and peripheral vascular structures in the distal part of the ascending aorta, and its effect on the coronary arteries and aortic valve in the proximal part of the ascending aorta. It should definitely be considered in elderly patients with hypertension in the presence of chest pain and unconsciousness. TEE is the chosen investigative method with high sensitivity and specificity in the diagnosis. It is important for a differential diagnosis during the pre-operative period and for determination of intra-operative treatment strategy.
